# Quality-checked species records from the German citizen science platform ArtenFinder

**DOI:** 10.3897/BDJ.13.e150687

**Published:** 2025-07-28

**Authors:** Thore Engel, Yannick Brenz, Hendrik Geyer, Jörg Holetschek, Aletta Bonn, Cathrina Balthasar, Susanne Bengsch, Romain J. G. Clément, Christian Dietzen, Chris Dlouhy, Jens Esser, Frederic Griesbaum, Matthias Haag, Karin-Simone Hauth, René Jarling, Stefan Kahlert, Norbert Kenntner, Jochen Krebühl, Julia Kruse, Steven Lischke, Robert Lücking, Lars Raphael Mayer, Susanne Müller, Thomas Nogatz, Michael Ochse, Sophie Ogan, Katharina C. M. von Oheimb, Parm Viktor von Oheimb, Gerrit A. A. Öhm, Korbinian Pacher, Manfred Alban Pfeifer, Charlotte Reutter, Oliver Röller, Frederik Rothe, Norbert S.N. Scheydt, Oliver Schmitz, Dominik Schmitz, Norman Wagner, Ulrike Willerding, Christoph Willigalla, Svea-Sophie Zimmermann, Martin Friedrichs-Manthey

**Affiliations:** 1 Friedrich-Schiller-Universität Jena, Fakultät für Biowissenschaften, Institut für Biodiversität, Jena, Germany Friedrich-Schiller-Universität Jena, Fakultät für Biowissenschaften, Institut für Biodiversität Jena Germany; 2 Helmholtz-Zentrum für Umweltforschung - UFZ, Department Biodiversität und Mensch, Leipzig, Germany Helmholtz-Zentrum für Umweltforschung - UFZ, Department Biodiversität und Mensch Leipzig Germany; 3 German Centre for Integrative Biodiversity Research (iDiv), Halle-Jena-Leipzig, Germany German Centre for Integrative Biodiversity Research (iDiv) Halle-Jena-Leipzig Germany; 4 Stiftung Naturschutz Berlin, Berlin, Germany Stiftung Naturschutz Berlin Berlin Germany; 5 Stiftung Natur und Umwelt Rheinland-Pfalz, Mainz, Germany Stiftung Natur und Umwelt Rheinland-Pfalz Mainz Germany; 6 Botanischer Garten und Botanisches Museum Berlin, Freie Universität Berlin, Berlin, Germany Botanischer Garten und Botanisches Museum Berlin, Freie Universität Berlin Berlin Germany; 7 Senckenberg Deutsches Entomologisches Institut, Müncheberg, Germany Senckenberg Deutsches Entomologisches Institut Müncheberg Germany; 8 Universität Potsdam, Institut für Biologie und Biochemie, Potsdam, Germany Universität Potsdam, Institut für Biologie und Biochemie Potsdam Germany; 9 ArtenFinder Rheinland-Pfalz, Mainz, Germany ArtenFinder Rheinland-Pfalz Mainz Germany; 10 Staatliche Vogelschutzwarte Rheinland-Pfalz, Landesamt für Umwelt, Mainz, Germany Staatliche Vogelschutzwarte Rheinland-Pfalz, Landesamt für Umwelt Mainz Germany; 11 ArtenFinder Berlin, Berlin, Germany ArtenFinder Berlin Berlin Germany; 12 Museum für Naturkunde – Leibniz Institute for Evolution and Biodiversity Science, Berlin, Germany Museum für Naturkunde – Leibniz Institute for Evolution and Biodiversity Science Berlin Germany; 13 Pfalzmuseum für Naturkunde - Pollichia-Museum, Bad Dürkheim, Germany Pfalzmuseum für Naturkunde - Pollichia-Museum Bad Dürkheim Germany; 14 Pollichia, Verein für Naturfoschung, Naturschutz und Umweltbildung e.V, Neustadt a.d. Weinstraße, Germany Pollichia, Verein für Naturfoschung, Naturschutz und Umweltbildung e.V Neustadt a.d. Weinstraße Germany; 15 Stabsstelle Klima, Boden, Biodiversität des Thünen-Institut, Braunschweig, Germany Stabsstelle Klima, Boden, Biodiversität des Thünen-Institut Braunschweig Germany; 16 Faculty of Life Sciences, Albrecht Daniel Thaer-Institute, Humboldt University of Berlin, Berlin, Germany Faculty of Life Sciences, Albrecht Daniel Thaer-Institute, Humboldt University of Berlin Berlin Germany; 17 Department of Biology and Ecology of Fishes, Leibniz-Institute for Freshwater Ecology and Inland Fischeries, Berlin, Germany Department of Biology and Ecology of Fishes, Leibniz-Institute for Freshwater Ecology and Inland Fischeries Berlin Germany; 18 Büro für Ökologische Gutachten, Bobenheim-Roxheim, Germany Büro für Ökologische Gutachten Bobenheim-Roxheim Germany; 19 BUND für Umwelt und Naturschutz Deutschland, Landesverband Rheinland-Pfalz e.V., Mainz, Germany BUND für Umwelt und Naturschutz Deutschland, Landesverband Rheinland-Pfalz e.V. Mainz Germany; 20 Institut für Naturkunde in Südwestdeutschland (NATUR SÜDWEST), Haßloch, Germany Institut für Naturkunde in Südwestdeutschland (NATUR SÜDWEST) Haßloch Germany; 21 Naturschutzverband Südpfalz, Herxheim, Germany Naturschutzverband Südpfalz Herxheim Germany; 22 Entomologische Gesellschaft ORION Berlin e.V., Berlin, Germany Entomologische Gesellschaft ORION Berlin e.V. Berlin Germany; 23 Zweckverband Natura Ill-Theel, Marpingen, Germany Zweckverband Natura Ill-Theel Marpingen Germany; 24 Willigalla Ökologische Gutachten, Mainz, Germany Willigalla Ökologische Gutachten Mainz Germany

**Keywords:** citizen science, biodiversity monitoring, volunteers, ArtenFinder, NFDI4Biodiversity, Berlin, Rhineland-Palatinate

## Abstract

**Background:**

Volunteers and citizen science initiatives play a crucial role for the documentation of species occurrences and distributions. When quality-checked and openly available, such data can provide information for biodiversity research and nature conservation. While some large international platforms reach a high number of opportunistic users around the world, there are also many smaller and regional citizen science initiatives, which often collaborate very closely with local authorities, conservation organisations and local experts and volunteers. Despite their high quality, data from such regional initiatives are often missing from global open data platforms, such as the Global Biodiversity Information Facility (GBIF).

**New information:**

Here, we present a quality-checked citizen science dataset published on GBIF with more than 1 million georeferenced species records with a geographic focus on the German federal states of Rhineland-Palatinate (Rheinland-Pfalz) and Berlin. The dataset originates from the collaborative observation platform ArtenFinder, which is run by the two federal states. While each state branch administers its own web platform, they share a common database. Users can upload, edit, manage and share their observations of animals, plants and fungi. Experts validate the species records, based on photographs and other media as well as on plausibility, which allows the data to be used by state authorities and for conservation management and research purposes. The data mobilisation and publication were enabled by the German National Research Data Infrastructure for Biodiversity (NFDI4Biodiversity) and the dataset is now also available through the Living Atlas of Nature Germany platform, a GBIF hosted portal.

## Introduction

Citizen science activities focused on biodiversity monitoring have surged over the past decade, significantly enhancing our understanding of species distributions and the dynamics of our changing biosphere (Chandler et al. 2017, Pocock et al. 2018, Stephenson and Stengel 2020). Citizen science projects are organised at different spatial scales, from small local conservation and monitoring projects to large global citizen science platforms, acting as social networks for nature observations (Richter et al. 2018). While many of the international players have crowd-sourced and published increasingly large amounts of open biodiversity data from around the globe (e.g. iNaturalist Contributors (2024), Observation.org (2024)), species records from many regional and small-scale citizen science initiatives remain relatively inaccessible to the broader scientific community (Engel et al. 2023). However, it is often especially the local and regional organisations that engage with and are trusted by local experts, authorities and volunteers, producing very targeted and high quality species occurrence data.

Despite the large volume of data generated by global citizen science initiatives, local nature conservation agencies in Germany and elsewhere make limited use of such widely-available data. Reasons for the low pick-up of citizenscience data include concerns over data quality and data validation and a lack of technical interfaces, hindering the incorporation of citizen science data into government databases and workflows (Owen and Parker 2018). At the same time, nature conservation agencies themselves have long relied on lay and expert volunteers for their own monitoring and species recording activities. While these government-held data are crucial for local conservation management, they are usually not readily available to the international research community and even local NGOs and citizens (Wetzel et al. 2018). Making data FAIR (i.e. findable, accessible, interoperable and reusable) is considered a major priority for research in general (Wilkinson et al. 2016) and participatory research and citizen science in particular (Hansen et al. 2021).

ArtenFinder is a regional citizen science platform in Germany, supported by the federal states of Rhineland-Palatinate (Rheinland-Pfalz) and Berlin and run by regional conservation organisations. The platform supports a very active community of lay and expert volunteers engaged in species recording, identification and validation. The data collected through the ArtenFinder system are largely opportunistic species occurrence data, which provide information for the work of the nature conservation agencies and non-government organisations in the two federal states. Here, we have mobilised the dataset to the Global Biodiversity Information Facility (GBIF) making it widely accessible to researchers around the world.

## General description

### Purpose

ArtenFinder was established in 2011 in Rhineland-Palatinate by a consortium of nature conservation NGOs (including Pollichia e. V., NABU|naturgucker and BUND) and the Ministry of the Environment Rhineland-Palatinate. In 2018, the Stiftung Naturschutz Berlin joined as project member. In 2020, the Stiftung Natur und Umwelt Rheinland-Pfalz assumed supervision of the platform, together with the Stiftung Naturschutz Berlin. The federal states of Hesse and North Rhine-Westphalia are associated partners.

The main purpose of ArtenFinder is to collect high quality species occurrence data for conservation purposes in Rhineland-Palatinate and Berlin as well as for faunistic and floristic research. ArtenFinder is managing a community of volunteers and a network of taxonomic experts, offering identification courses, excursions and community events (e.g. recording events for invasive species). Additionally, ArtenFinder seeks to deepen the public's connection with nature by providing accessible information on local species occurrences and fostering an appreciation for biodiversity in their immediate surroundings.

At the heart of the project are the ArtenFinder recording portals of Rhineland-Palatinate (https://artenfinder.rlp.de/) and Berlin (https://berlin.artenfinder.net/) and the ArtenFinder web app (https://artenfinder.net/artenfinder-pwa/). They are available in German language and enable registered users to upload, edit, manage and share their species observations. Observations from both federal states feed into a shared database, which is connected to the states' nature conservation agencies. Furthermore, validated data are published to GBIF through the BioCASe Provider Software (i.e. Biological Collections Access Service, https://www.biocase.org/products/provider_software). Additionally, the data are now also available in the national biodiversity portal Lebendiger Atlas der Natur Deutschlands, which is a GBIF-hosted portal for species occurrence data. The data mobilisation to GBIF was enabled by the German National Research Data Infrastructure for Biodiversity (NFDI4Biodiversity). All figures and numbers in this manuscript reflect the dataset as of 31 December 2024, which amounts to more than a million records.

## Sampling methods

### Sampling description

**Figure 1.**
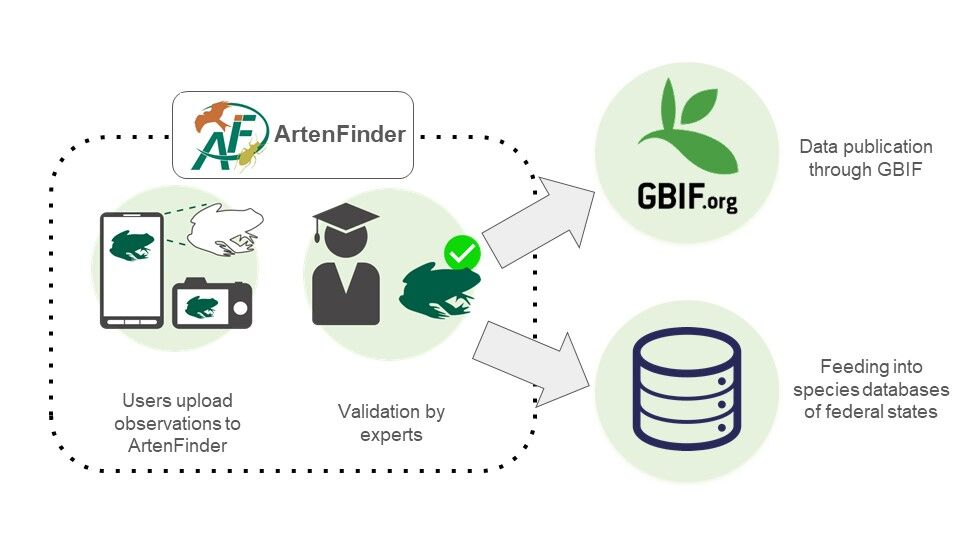
ArtenFinder data pipeline from users to expert validation to integration in GBIF and federal state authorities databases.

The large majority of records in this dataset consists of opportunistic species occurrence data collected by volunteers through the ArtenFinder portal (Fig. 1). The minimum data requirements for species observations submitted to ArtenfFinder are spatial coordinates and a date. In addition, it is possible and strongly recommended to provide pictures, sound files and more detailed descriptions of the specific observation. The system also allows to record information such as life stage, breeding status and the number of individuals. However, this information is currently not published to GBIF. Aside from the opportunistic citizen science data, the ArtenFinder datasets also include a smaller number of species records from more systematic and professional monitoring activities. This includes data from natural history societies (e.g. Arbeitskreis Heimische Orchideen) and professional monitoring activities carried out by the organisations directly involved on the ArtenFinder Project (e.g. European hamster monitoring carried out by Stiftung Naturschutz RLP).

In most cases, the dataset provides exact coordinates for records. However, users can flag observations as sensitive and all records of threatened or rare species are automatically marked as sensitive within the ArtenFinder system. For these records, locations are shared as the centroid of a 5 km × 5 km grid cell containing the sensitive observation.

### Quality control

Since most of the data are collected by untrained volunteers and the main purpose of the collected data is to provide information for federal nature conservation purposes, quality control is a high priority in the ArtenFinder project. A group of more than 50 taxonomic experts are involved in data validation based on photographs and other media as well as on the plausibility of records. To become an expert for a taxonomic group, a proof of taxonomic expertise is necessary. A proof can, for example, be a relevant scientific career, peer-reviewed publications on the taxonomic group in question, membership in a relevant natural history society or simply being a well-respected expert for the given taxon.

Although providing a photo or sound file is not mandatory for reporting a species observation to ArtenFinder, data validation heavily relies on such media. Consequently, records lacking photo or sound evidence are rarely included in the validated dataset presented here. Exceptions are made for records submitted by users with demonstrable taxonomic expertise for the species in question (e.g. more than five previously validated records of the same species). Furthermore, users can evidence their records by providing a written description of their sightings, highlighting the characteristics that distinguish the recorded species from similar species.

## Geographic coverage

### Description

**Figure 2.**
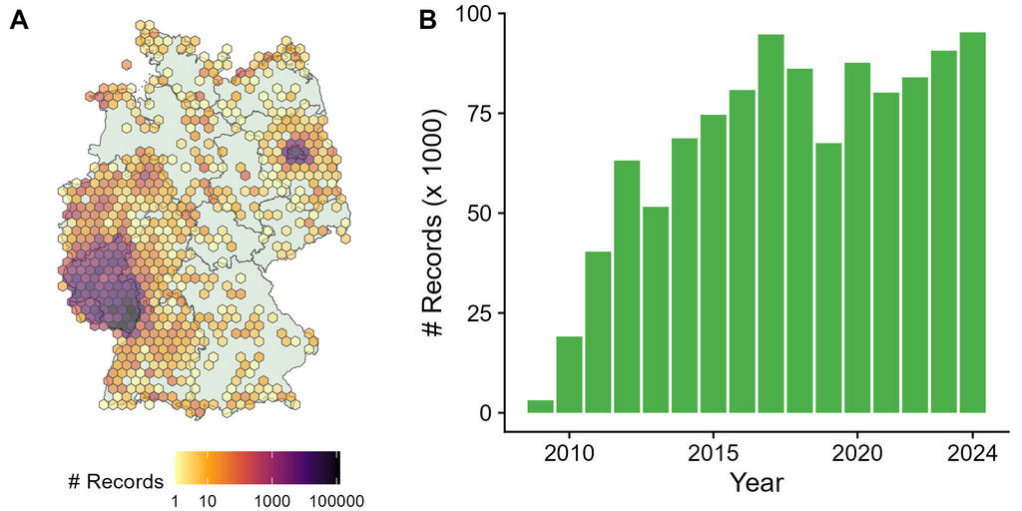
Geographic and temporal scope of the ArtenFinder dataset. **A** Spatial distribution of the records in Germany, based on a 20 km hexagonal grid. Records from outside of Germany are omitted in this figure (i.e. < 1% of data); **B** Number of records per year. Records from before 2009 are omitted in this figure (i.e. < 1% of data).

While it is generally possible to submit species records from around the globe to ArtenFinder, more than 99% of the records are from Germany. The majority of recording sites are located in the State of Rhineland-Palatinate and surroundings and a smaller number in and around the State of Berlin, where the project started later and which covers a smaller area. Within Rhineland-Palatinate, there is an imbalance with more records in the southern parts than in the northern parts. Additional records are scattered in the rest of Germany; however, most data are from the southwest of the country (Fig. 2A).

## Taxonomic coverage

### Description

**Figure 3.**
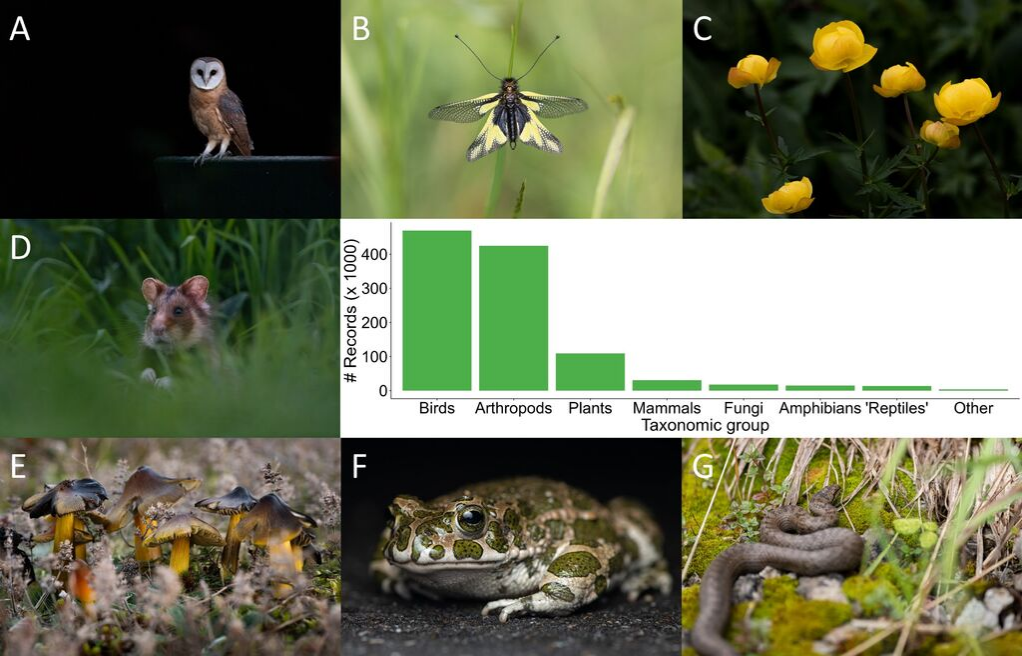
Taxonomic distribution of the ArtenFinder dataset with exemplary photos, representing: **A** birds (*Tytoalba*); **B** arthropods (*Libelloidescoccajus*); **C** plants (*Trolliuseuropaeus*); **D** mammals (*Cricetuscricetus*); **E** fungi including lichens (*Hygrocybeconica*); **F** amphibians (*Bufotesviridis*); **G** "reptiles" (*Coronellaaustriaca*). Photo credits: Chris Dlouhy

The dataset has a very broad taxonomic scope, reflecting the species observations of the ArtenFinder users. However, most observations are on bird and arthropod species, with a smaller share of other animal taxa, plants and fungi (including lichens) (Fig. 3). Table 1 summarises the taxonomic coverage of the dataset at the order level, including the number of records and the number of recorded species per order.

## Temporal coverage

### Notes

While more than 99% of the data were recorded after the launch of the project in 2011, the dataset contains a small number of records from earlier years (Fig. 2B). This is because retroactive data entry is also possible and there have been some data imports of previously existing records. The data collection is still ongoing and the ArtenFinder dataset on GBIF is updated weekly.

## Usage licence

### Usage licence

Creative Commons Public Domain Waiver (CC-Zero)

## Data resources

### Data package title

ArtenFinder dataset on GBIF

### Resource link

https://doi.org/10.15468/jnnald

### Alternative identifiers

https://artenfinder.rlp.de/biocase/pywrapper.cgi?dsa=artenfinder; https://www.gbif.org/dataset/aa6c5ee6-d4d7-4a65-a04f-379cffbf4842

### Number of data sets

1

### Data set 1.

#### Data set name

ArtenFinder dataset on GBIF

#### Data format

DarwinCore Archive

#### Character set

UTF-8

#### Download URL

https://doi.org/10.15468/dl.77k8wp

#### Description

This is the ArtenFinder dataset available through GBIF. The dataset is provided to GBIF through the BioCASe Provider Software using the ABCD data standard (Access to Biological Collections Data). The ABCD XML archive harvested by GBIF is refreshed weekly. The download link above reflects the static dataset at the time of writing this manuscript (17.02.2025). The resource link directs to the dynamic dataset on GBIF.

Since GBIF maps ABCD elements to the DarwinCore standard and since the GBIF download comprises a DarwinCore archive, the respective DarwinCore terms are given below. Only data items provided by ArtenFinder are listed here, not fields inferred and added by GBIF (e.g. higher taxonomy, administrative areas etc.).

**Data set 1.** 

Column label	Column description
occurrenceID	A globally unique identifier for the observation.
catalogNumber	A unique identifier within the dataset; same as occurrenceID.
institutionCode	The name in use by the institution having custody of the object(s) or information referred to in the record.
collectionCode	The name identifying the dataset from which the record was derived.
references	A related resource that is referenced by the described record. In our case, it is a link to a machine-readable representation of the observation serialised as XML.
license	A legal document giving official permission to do something with the resource. ArtenFinder publishes data under a Creative Commons 1.0 Public Domain Dedication licence (http://creativecommons.org/publicdomain/zero/1.0/).
basisOfRecord	The specific nature of the data record; for ArtenFinder, it is "HumanObservation".
occurrenceStatus	A statement about the presence or absence of the taxon at the given location; for all ArtenFinder records, it is "present".
eventDate	The date at which the organism has been observed.
decimalLongitude	The geographic longitude (in decimal degrees, using the spatial reference system given in geodeticDatum) of the observation.
decimalLatitude	The geographic latitude (in decimal degrees, using the spatial reference system given in geodeticDatum) of the observation.
geodeticDatum	The ellipsoid, geodetic datum or spatial reference system (SRS), upon which the geographic coordinates given in decimalLatitude and decimalLongitude are based. In our case, it is "WGS84". Within the DarwinCore archive, this information is stored in the file meta.xml.
dateIdentified	The date on which the organism was determined as representing the taxon.
scientificName	The full scientific name of the taxon including at least genus name and species epithet and, in some cases, including a subspecies epithet or variety. For some records of the ArtenFinder dataset, this field also contains species aggregates or taxonomic names that are not resolved at the species level.
coordinateUncertaintyInMeters	The horizontal distance (in metres) from the given decimalLatitude and decimalLongitude describing the smallest circle containing the whole of the location. Sensitive ArtenFinder records are shared as centroids of a 5 km x 5 km grid cell, corresponding to a value of 3536.

## Additional information

The authors acknowledge that there are also other well-established species recording platforms and citizen-science apps available in the study area, some of which are more targeted to specific taxa or user communities (e.g. Ornitho for birds). Many ArtenFinder users and experts are active on many of these platforms and there are ongoing efforts to establish data flows between ArtenFinder and other initiatives with open data policies (e.g. NABU|naturgucker and Observation.org). These imported data, however, are not and will not be part of the GBIF ArtenFinder dataset described here, as they are already published by the original data holders.

## Figures and Tables

**Table 1.** Taxonomic coverage of the dataset at the order level with number of records and recorded species per order. The taxonomy was matched against the GBIF taxonomic backbone (some cells are left blank for this reason). Note that the table is ordered alphabetically.

**Kingdom**	**Phylum**	**Class**	**Order**	# **Records**	# **Species**
Animalia	Annelida	Clitellata	Arhynchobdellida	1	1
Animalia	Annelida	Clitellata	Crassiclitellata	2	1
Animalia	Arthropoda	Arachnida	Araneae	7211	177
Animalia	Arthropoda	Arachnida	Ixodida	95	3
Animalia	Arthropoda	Arachnida	Opiliones	252	13
Animalia	Arthropoda	Arachnida	Pseudoscorpiones	7	1
Animalia	Arthropoda	Arachnida	Trombidiformes	51	4
Animalia	Arthropoda	Branchiopoda	Anostraca	1	1
Animalia	Arthropoda	Branchiopoda	Notostraca	3	1
Animalia	Arthropoda	Chilopoda	Lithobiomorpha	43	1
Animalia	Arthropoda	Chilopoda	Scutigeromorpha	103	1
Animalia	Arthropoda	Diplopoda	Glomerida	31	1
Animalia	Arthropoda	Diplopoda	Julida	8	1
Animalia	Arthropoda	Diplopoda	Polyxenida	2	1
Animalia	Arthropoda	Insecta	Archaeognatha	2	1
Animalia	Arthropoda	Insecta	Blattodea	477	7
Animalia	Arthropoda	Insecta	Coleoptera	39288	1604
Animalia	Arthropoda	Insecta	Dermaptera	272	5
Animalia	Arthropoda	Insecta	Diptera	3058	148
Animalia	Arthropoda	Insecta	Ephemeroptera	3	2
Animalia	Arthropoda	Insecta	Hemiptera	15071	436
Animalia	Arthropoda	Insecta	Hymenoptera	14828	311
Animalia	Arthropoda	Insecta	Lepidoptera	269017	1493
Animalia	Arthropoda	Insecta	Mantodea	2791	1
Animalia	Arthropoda	Insecta	Mecoptera	116	4
Animalia	Arthropoda	Insecta	Neuroptera	140	10
Animalia	Arthropoda	Insecta	Odonata	52034	66
Animalia	Arthropoda	Insecta	Orthoptera	20088	64
Animalia	Arthropoda	Insecta	Psocodea	2	1
Animalia	Arthropoda	Insecta	Raphidioptera	6	3
Animalia	Arthropoda	Insecta	Trichoptera	14	13
Animalia	Arthropoda	Insecta	Zygentoma	10	2
Animalia	Arthropoda	Malacostraca	Decapoda	245	9
Animalia	Arthropoda	Malacostraca	Isopoda	13	4
Animalia	Chordata		Anguilliformes	19	1
Animalia	Chordata		Cypriniformes	343	26
Animalia	Chordata		Cyprinodontiformes	2	1
Animalia	Chordata		Esociformes	56	1
Animalia	Chordata		Gadiformes	1	1
Animalia	Chordata		Gasterosteiformes	47	2
Animalia	Chordata		Perciformes	99	7
Animalia	Chordata		Salmoniformes	55	5
Animalia	Chordata		Scorpaeniformes	34	1
Animalia	Chordata		Siluriformes	6	2
Animalia	Chordata	Amphibia	Anura	10358	16
Animalia	Chordata	Amphibia	Caudata	4514	5
Animalia	Chordata	Aves	Accipitriformes	31969	18
Animalia	Chordata	Aves	Anseriformes	35681	49
Animalia	Chordata	Aves	Apodiformes	2242	1
Animalia	Chordata	Aves	Bucerotiformes	766	1
Animalia	Chordata	Aves	Caprimulgiformes	44	1
Animalia	Chordata	Aves	Charadriiformes	6208	55
Animalia	Chordata	Aves	Ciconiiformes	5782	2
Animalia	Chordata	Aves	Columbiformes	19487	5
Animalia	Chordata	Aves	Coraciiformes	3443	2
Animalia	Chordata	Aves	Cuculiformes	2549	1
Animalia	Chordata	Aves	Falconiformes	9884	5
Animalia	Chordata	Aves	Galliformes	5504	4
Animalia	Chordata	Aves	Gaviiformes	16	4
Animalia	Chordata	Aves	Gruiformes	13506	7
Animalia	Chordata	Aves	Otidiformes	1	1
Animalia	Chordata	Aves	Passeriformes	278987	113
Animalia	Chordata	Aves	Pelecaniformes	14892	12
Animalia	Chordata	Aves	Phoenicopteriformes	1	1
Animalia	Chordata	Aves	Piciformes	21014	7
Animalia	Chordata	Aves	Podicipediformes	4476	5
Animalia	Chordata	Aves	Psittaciformes	1313	2
Animalia	Chordata	Aves	Strigiformes	2110	9
Animalia	Chordata	Aves	Suliformes	5351	3
Animalia	Chordata	Mammalia	Artiodactyla	5426	5
Animalia	Chordata	Mammalia	Carnivora	5216	13
Animalia	Chordata	Mammalia	Chiroptera	2197	21
Animalia	Chordata	Mammalia	Erinaceomorpha	921	1
Animalia	Chordata	Mammalia	Lagomorpha	3640	2
Animalia	Chordata	Mammalia	Rodentia	12694	21
Animalia	Chordata	Mammalia	Soricomorpha	620	9
Animalia	Chordata	Petromyzonti	Petromyzontiformes	12	1
Animalia	Chordata	Squamata		13065	10
Animalia	Chordata	Testudines		659	12
Animalia	Mollusca	Bivalvia	Sphaeriida	2	1
Animalia	Mollusca	Bivalvia	Unionida	5	4
Animalia	Mollusca	Bivalvia	Venerida	11	2
Animalia	Mollusca	Gastropoda		49	4
Animalia	Mollusca	Gastropoda	Architaenioglossa	2	1
Animalia	Mollusca	Gastropoda	Cycloneritida	1	1
Animalia	Mollusca	Gastropoda	Ellobiida	4	1
Animalia	Mollusca	Gastropoda	Littorinimorpha	2	1
Animalia	Mollusca	Gastropoda	Stylommatophora	2614	49
Bacteria	Cyanobacteria	Cyanobacteriia	Cyanobacteriales	1	1
Chromista	Oomycota	Peronosporea	Peronosporales	12	11
Fungi	Ascomycota	Dothideomycetes		2	2
Fungi	Ascomycota	Dothideomycetes	Botryosphaeriales	1	1
Fungi	Ascomycota	Dothideomycetes	Capnodiales	2	2
Fungi	Ascomycota	Dothideomycetes	Dothideales	1	1
Fungi	Ascomycota	Dothideomycetes	Mycosphaerellales	14	12
Fungi	Ascomycota	Dothideomycetes	Pleosporales	4	2
Fungi	Ascomycota	Dothideomycetes	Venturiales	3	3
Fungi	Ascomycota	Eurotiomycetes	Eurotiales	5	2
Fungi	Ascomycota	Geoglossomycetes	Geoglossales	56	5
Fungi	Ascomycota	Laboulbeniomycetes	Laboulbeniales	5	1
Fungi	Ascomycota	Lecanoromycetes	Baeomycetales	4	1
Fungi	Ascomycota	Lecanoromycetes	Caliciales	4	3
Fungi	Ascomycota	Lecanoromycetes	Lecanorales	94	26
Fungi	Ascomycota	Lecanoromycetes	Peltigerales	2	2
Fungi	Ascomycota	Lecanoromycetes	Teloschistales	10	2
Fungi	Ascomycota	Leotiomycetes	Helotiales	195	69
Fungi	Ascomycota	Leotiomycetes	Leotiales	37	2
Fungi	Ascomycota	Leotiomycetes	Phacidiales	31	1
Fungi	Ascomycota	Leotiomycetes	Rhytismatales	34	3
Fungi	Ascomycota	Pezizomycetes	Pezizales	565	58
Fungi	Ascomycota	Sordariomycetes	Diaporthales	5	4
Fungi	Ascomycota	Sordariomycetes	Hypocreales	39	7
Fungi	Ascomycota	Sordariomycetes	Microascales	1	1
Fungi	Ascomycota	Sordariomycetes	Phyllachorales	3	1
Fungi	Ascomycota	Sordariomycetes	Xylariales	130	12
Fungi	Ascomycota	Taphrinomycetes	Taphrinales	1	1
Fungi	Basidiomycota	Agaricomycetes		30	1
Fungi	Basidiomycota	Agaricomycetes	Agaricales	7677	505
Fungi	Basidiomycota	Agaricomycetes	Amylocorticiales	18	2
Fungi	Basidiomycota	Agaricomycetes	Auriculariales	230	8
Fungi	Basidiomycota	Agaricomycetes	Boletales	3514	93
Fungi	Basidiomycota	Agaricomycetes	Cantharellales	658	16
Fungi	Basidiomycota	Agaricomycetes	Geastrales	343	17
Fungi	Basidiomycota	Agaricomycetes	Gloeophyllales	92	6
Fungi	Basidiomycota	Agaricomycetes	Gomphales	50	12
Fungi	Basidiomycota	Agaricomycetes	Hymenochaetales	138	21
Fungi	Basidiomycota	Agaricomycetes	Hysterangiales	1	1
Fungi	Basidiomycota	Agaricomycetes	Phallales	240	6
Fungi	Basidiomycota	Agaricomycetes	Polyporales	1947	74
Fungi	Basidiomycota	Agaricomycetes	Russulales	1006	109
Fungi	Basidiomycota	Agaricomycetes	Thelephorales	30	6
Fungi	Basidiomycota	Dacrymycetes	Dacrymycetales	154	7
Fungi	Basidiomycota	Exobasidiomycetes	Microstromatales	5	2
Fungi	Basidiomycota	Microbotryomycetes	Microbotryales	4	3
Fungi	Basidiomycota	Pucciniomycetes	Pucciniales	99	57
Fungi	Basidiomycota	Tremellomycetes	Tremellales	136	2
Fungi	Basidiomycota	Ustilaginomycetes	Ustilaginales	4	2
Fungi	Mucoromycota	Mucoromycetes	Mucorales	2	1
Plantae	Bryophyta	Bryopsida	Archidiales	3	1
Plantae	Bryophyta	Bryopsida	Aulacomniales	107	2
Plantae	Bryophyta	Bryopsida	Bartramiales	6	3
Plantae	Bryophyta	Bryopsida	Bryales	51	13
Plantae	Bryophyta	Bryopsida	Buxbaumiales	52	2
Plantae	Bryophyta	Bryopsida	Dicranales	207	25
Plantae	Bryophyta	Bryopsida	Encalyptales	1	1
Plantae	Bryophyta	Bryopsida	Funariales	2	2
Plantae	Bryophyta	Bryopsida	Grimmiales	22	8
Plantae	Bryophyta	Bryopsida	Hedwigiales	1	1
Plantae	Bryophyta	Bryopsida	Hookeriales	6	1
Plantae	Bryophyta	Bryopsida	Hypnales	456	61
Plantae	Bryophyta	Bryopsida	Orthotrichales	75	19
Plantae	Bryophyta	Bryopsida	Pottiales	77	27
Plantae	Bryophyta	Bryopsida	Splachnales	2	1
Plantae	Bryophyta	Polytrichopsida	Polytrichales	155	7
Plantae	Bryophyta	Polytrichopsida	Tetraphidales	8	1
Plantae	Bryophyta	Sphagnopsida	Sphagnales	1376	25
Plantae	Charophyta	Charophyceae	Charales	1	1
Plantae	Marchantiophyta	Jungermanniopsida	Jungermanniales	61	27
Plantae	Marchantiophyta	Jungermanniopsida	Metzgeriales	23	5
Plantae	Marchantiophyta	Jungermanniopsida	Pallaviciniales	5	1
Plantae	Marchantiophyta	Jungermanniopsida	Pelliales	6	1
Plantae	Marchantiophyta	Jungermanniopsida	Porellales	40	5
Plantae	Marchantiophyta	Jungermanniopsida	Ptilidiales	1	1
Plantae	Marchantiophyta	Marchantiopsida	Lunulariales	1	1
Plantae	Marchantiophyta	Marchantiopsida	Marchantiales	24	5
Plantae	Tracheophyta	Ginkgoopsida	Ginkgoales	2	1
Plantae	Tracheophyta	Liliopsida	Acorales	9	1
Plantae	Tracheophyta	Liliopsida	Alismatales	905	32
Plantae	Tracheophyta	Liliopsida	Asparagales	17783	101
Plantae	Tracheophyta	Liliopsida	Commelinales	1	1
Plantae	Tracheophyta	Liliopsida	Dioscoreales	2	2
Plantae	Tracheophyta	Liliopsida	Liliales	1380	10
Plantae	Tracheophyta	Liliopsida	Poales	8399	217
Plantae	Tracheophyta	Lycopodiopsida	Lycopodiales	77	6
Plantae	Tracheophyta	Magnoliopsida	Apiales	2710	62
Plantae	Tracheophyta	Magnoliopsida	Aquifoliales	135	1
Plantae	Tracheophyta	Magnoliopsida	Asterales	14548	210
Plantae	Tracheophyta	Magnoliopsida	Boraginales	1354	34
Plantae	Tracheophyta	Magnoliopsida	Brassicales	3287	87
Plantae	Tracheophyta	Magnoliopsida	Buxales	21	1
Plantae	Tracheophyta	Magnoliopsida	Caryophyllales	5624	131
Plantae	Tracheophyta	Magnoliopsida	Celastrales	215	3
Plantae	Tracheophyta	Magnoliopsida	Ceratophyllales	125	2
Plantae	Tracheophyta	Magnoliopsida	Cornales	258	6
Plantae	Tracheophyta	Magnoliopsida	Cucurbitales	119	3
Plantae	Tracheophyta	Magnoliopsida	Dipsacales	1845	32
Plantae	Tracheophyta	Magnoliopsida	Ericales	3592	36
Plantae	Tracheophyta	Magnoliopsida	Fabales	5227	96
Plantae	Tracheophyta	Magnoliopsida	Fagales	2073	19
Plantae	Tracheophyta	Magnoliopsida	Gentianales	2256	35
Plantae	Tracheophyta	Magnoliopsida	Geraniales	1078	15
Plantae	Tracheophyta	Magnoliopsida	Lamiales	8330	184
Plantae	Tracheophyta	Magnoliopsida	Laurales	1	1
Plantae	Tracheophyta	Magnoliopsida	Magnoliales	6	1
Plantae	Tracheophyta	Magnoliopsida	Malpighiales	2808	70
Plantae	Tracheophyta	Magnoliopsida	Malvales	659	23
Plantae	Tracheophyta	Magnoliopsida	Myrtales	728	21
Plantae	Tracheophyta	Magnoliopsida	Nymphaeales	205	3
Plantae	Tracheophyta	Magnoliopsida	Oxalidales	222	4
Plantae	Tracheophyta	Magnoliopsida	Piperales	81	2
Plantae	Tracheophyta	Magnoliopsida	Proteales	6	1
Plantae	Tracheophyta	Magnoliopsida	Ranunculales	5566	78
Plantae	Tracheophyta	Magnoliopsida	Rosales	6786	121
Plantae	Tracheophyta	Magnoliopsida	Santalales	298	6
Plantae	Tracheophyta	Magnoliopsida	Sapindales	2358	14
Plantae	Tracheophyta	Magnoliopsida	Saxifragales	975	28
Plantae	Tracheophyta	Magnoliopsida	Solanales	598	20
Plantae	Tracheophyta	Magnoliopsida	Vitales	23	5
Plantae	Tracheophyta	Pinopsida	Pinales	722	12
Plantae	Tracheophyta	Polypodiopsida	Equisetales	319	8
Plantae	Tracheophyta	Polypodiopsida	Hymenophyllales	1	1
Plantae	Tracheophyta	Polypodiopsida	Ophioglossales	17	2
Plantae	Tracheophyta	Polypodiopsida	Osmundales	151	1
Plantae	Tracheophyta	Polypodiopsida	Polypodiales	2302	30
Plantae	Tracheophyta	Polypodiopsida	Salviniales	35	3
Protozoa	Mycetozoa	Myxomycetes	Cribrariales	100	4
Protozoa	Mycetozoa	Myxomycetes	Physarales	77	4
Protozoa	Mycetozoa	Myxomycetes	Stemonitidales	2	2
Protozoa	Mycetozoa	Myxomycetes	Trichiales	3	1
Protozoa	Mycetozoa	Protosteliomycetes	Ceratiomyxales	3	1
